# Combined TaTME with SP Robot for Low Anterior Resection in Rectal Cancer: rSPa TaTME

**DOI:** 10.3390/cancers17081328

**Published:** 2025-04-15

**Authors:** Nouran O. Keshk, Mauricio E. Perez-Pachon, Ibrahim Gomaa, Sara Aboelmaaty, David W. Larson, Kristen K. Rumer, Sherief F. Shawki

**Affiliations:** 1 Division of Colorectal Surgery, Department of Surgery, Mayo Clinic, Rochester, MN 55905, USA; keshk.nouran@mayo.edu (N.O.K.); perezpachon.mauricio@mayo.edu (M.E.P.-P.); gomaa.ibrahim@mayo.edu (I.G.); aboelmaaty.saraahmadmoha@mayo.edu (S.A.); larson.david2@mayo.edu (D.W.L.); 2 Division of Colon and Rectal Surgery, Department of Surgery, Mayo Clinic, Rochester, MN 55905, USA

**Keywords:** robotic surgery, rectal cancer, transanal mesorectal excision, colorectal

## Abstract

**Background**: Total mesorectal excision (TME) remains the gold standard for the resection of rectal cancer regardless of the modality: open, laparoscopic, or robotic. The transanal TME (TaTME) approach has been utilized to overcome the difficulties encountered with the dissection of tumors in the distal pelvis. Recently, a single-port robotic approach (rSPa) was introduced, where three arms and a camera emanate from a 2.5 cm diameter port. This report presents the first experience in the United States combining those two approaches (rSPa TaTME) in rectal cancer, evaluating its safety and oncologic outcomes. **Methods**: This is a retrospective review of our prospectively maintained rectal cancer database. Patient demographics, tumor characteristics, neoadjuvant treatment, and oncologic and surgical outcomes were recorded. **Results**: Between May 2022 and August 2024, ten patients (six females, four males) with a median age at surgery of 53 years (range: 38–85) and a mean BMI of 26 (±5) kg/m^2^ were included for analysis. The median distance of tumors from the anorectal junction was 3.2 cm (range: 2–5.3 cm). All patients had negative margins, with eight complete TME specimens, one near complete, and one incomplete. The mean number of lymph nodes harvested was 24 (±11). The average operative time was 351 (243–411) min. The average length of stay was four days. The ileostomy was reversed in nine out of ten patients. Six patients experienced complications within 30 days of surgery. There were no local or distal recurrences, with a mean follow-up of 20 months (range: 4–30). **Conclusions**: rSPa TaTME is a unique and innovative method of combining two minimally advanced approaches for the resection of distal rectal cancers, with acceptable surgical and oncologic outcomes.

## Simple Summary

Transanal total mesorectal excision (TaTME) has been utilized to overcome the difficulties associated with distal proctectomy. At the same time, single-port robotic abdominal (rSPa) procedures allow minimally invasive, single-incision abdominal dissection during proctectomy (rSPa TaTME). We are reporting our initial experience with ten patients who underwent combined single-port robotic abdominal and TaTME approaches for the resection of distal rectal cancers. The benefits of this approach are that (1) the defunctionalizing stoma is placed at the SP site, so there is no appearance of incisions, and (2) the TaTME approach facilitates distal TME and anastomosis after the resection of very distal tumors, even in anatomically challenging pelvises, while maintaining oncologic principles. As such, rSPa TaTME can be considered a safe and feasible approach with appropriate experience, demonstrating good medium-term oncological and surgical outcomes.

## 1. Introduction

Rectal cancer treatment has evolved significantly over the past few decades, with surgical resection remaining a cornerstone of curative therapy. Among the various surgical approaches, total mesorectal excision (TME) has become the gold standard since its introduction in the early 1980s [1]. The TME approach involves the precise removal of the rectum and mesorectum, reducing local recurrence rates and improving overall survival [2,3,4,5]. Recent advancements in minimally invasive surgery, particularly robotic-assisted TME, have refined the procedure [6,7,8]. Minimally invasive approaches offer the benefits of reduced postoperative pain, shorter hospital stays, and faster recovery while maintaining oncological efficacy [9,10]. Robotic-assisted surgery for rectal cancer provides advantages over both open and laparoscopic surgery in terms of patient outcomes and reducing postoperative complications [11,12,13,14,15,16,17,18]. The single-port robotic abdominal approach (rSPa) for rectal cancer offers additional benefits compared to traditional multi-port robotic surgery or single-incision laparoscopic surgery, including shorter hospital stays, reduced operative times, lower pain, faster recovery, and improved cosmetic results, while maintaining oncologic outcomes [19,20]. For distal tumors, transanal total mesorectal excision (TaTME) facilitates a “bottom-up” dissection approach, which is particularly advantageous in patients with narrow pelvises. Transanal TME allows for excellent visualization of the distal anatomy to improve resection completeness and facilitates anastomosis in patients for whom an abdominoperineal resection might otherwise be required [21], with safe oncologic outcomes [22].

Combining the multi-port robotic abdominal and TaTME approaches for proctectomy has shown advantages in oncological safety and functional preservation EI [23,24,25,26]. By combining the rSPa approach for the abdominal portion of the proctectomy with TaTME for distal rectal cancers, patients have no abdominal incisions other than the SP site, which is used for refunctioning ileostomy. This hybrid approach has the potential to give patients the benefits of less pain, shorter hospital stays, and improved aesthetic outcomes while maintaining the oncologic outcomes of the resection of distal rectal tumors. Here, we report our experience using a combined rSPa and TaTME approach for rectal cancer resection in ten patients.

## 2. Materials and Methods

This is a descriptive report presenting the safety and feasibility of a new minimally invasive surgical approach combining transanal total mesorectal excision (TaTME) and a single-port (SP) robotic platform for managing distal resectable rectal cancer. We retrospectively reviewed our prospectively maintained database of patients undergoing rectal cancer resection. We evaluated the records from our initial ten patients who underwent rSPa TaTME from May 2022 to August 2024. The variables analyzed included demographics, pre- and post-operative tumor staging, tumor characteristics (including size and distance from the tumor’s distal margin to the anorectal junction), neoadjuvant and adjuvant therapy (chemoradiotherapy and/or radiotherapy), pathologic criteria (including resection completeness, resection margin status, and response to neoadjuvant treatment), the number of lymph nodes harvested, the average operative time, whether ileostomy was reversed, mean follow-up duration, recurrence rates, and 30-day postoperative morbidity. The descriptive statistics are presented as frequencies (percentages) for categorical variables and the mean ± standard deviation (SD) or median (interquartile range) for quantitative variables. All statistical analyses were conducted using SPSS^®^ (version 28; SPSS^®^, Inc., Armonk, NY, USA).

### 2.1. Surgical Technique and Operative Room Organization

The procedure consisted of 4 phases. The first was the perineal (TaTME) portion, followed by the abdominal portion. Subsequently, the specimen was retracted, usually transanally, and anastomosis was performed. Finally, a diverting loop ileostomy was created where the SP robot was docked. A drain was placed through an assistant AirSeal port, 8 mm. In 2 cases, where an en-bloc total abdominal hysterectomy was performed, the specimen was extracted through the stoma aperture and the anvil was placed in the colonic conduit prior to replacing the conduit in the abdominal cavity.

**Figure 1:**
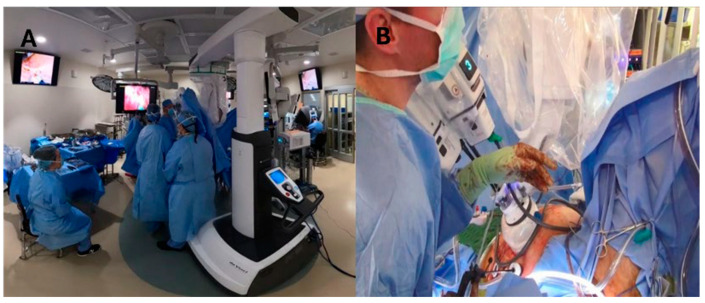
(**A**) Operative room set up. (**B**) Simultaneous team approach.

The patient was placed in the lithotomy position with both arms tucked at their sides to allow for simultaneous abdominal and transanal access. When the dual/simultaneous team approach was complete, the transperineal (TaTME) portion was commenced first, continuing until the desired surgical plane was reached. Subsequently, the stoma aperture was created, followed by docking the robot after positioning the operative table in the Trendelenburg position with a tilt to the right (Figure 1).

#### 2.1.1. Perineal Phase

**Figure 2:**
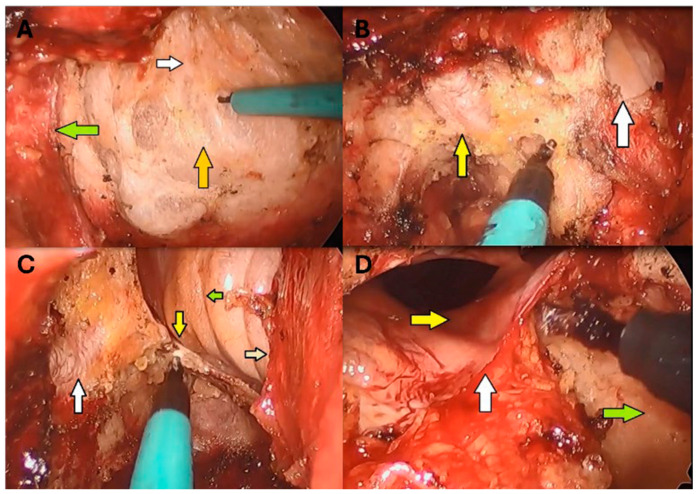
(**A**) posterior dissection at the level of Fascia of Waldeyer’s (recto-sacral fascia). White arrow: posterior rectum. Orange arrow: shinny white fascia of Waldeyer’s (recto-sacral fascia). Green arrow: right distal pelvic side wall. (**B**) Right side pelvic dissection till the level of the peritoneal reflection which shown already divided. Yellow arrow: right side neurovascular bundle. White arrow: divided peritoneal reflection. The cautery tip is dividing the right lateral portion of the peritoneal reflection. (**C**) The right lateral part is fully freed. White arrow: neurovascular bundle fully lateralized. Yellow arrow: right pararectal gutter. Green arrow: upper rectum right side. Grey arrow: divided peritoneal reflection. (**D**) Same lateral dissection on the left side. Yellow arrow: left pararectal gutter. White arrow: divided peritoneal reflection. Green arrow: lateralized neurovascular bundle on the left side.

This portion commenced with placing anal effacement sutures to open the anus, as an alternative to other commercially available retractors. After gentle and gradual dilation of the anal canal, the access channel was placed inside it. An occlusive purse-string suture was placed in the rectum distal to the location of the tumor using 0 Prolene (Ethicon Inc., Somerville, NJ, USA) on an SH needle. Betadine (Atlantis Consumer Healthcare Inc., Bridgewater, NJ, USA) solution was applied to the distal rectum. The gel cap was assembled after placing 2 ports and an 8 mm AirSeal (ConMed Corporation, Largo, FL, USA) port in a triangular formation. Insufflation was established using the AirSeal. A full-thickness circumferential proctectomy was performed, with the TME plane entered posterolaterally and then connected at the midline. Subsequently, dissection was extended anteriorly, and the plane between the anorectal junction and the prostate/vagina was identified. Dissection was then resumed posteriorly, extending cephalad and then widening the dissection bilaterally toward the distal pelvic wall. At the level of S3, Waldeyer’s (rectosacral) fascia was incised sharply. Then, the dissection trajectory was directed upward, near a right angle and parallel to the upper sacrum, at the S1/S2 level. At this point, attention was turned toward the anterior plane, which had been initially identified. Dissection then ensued cephalad to the level of the peritoneal reflection, which was incised sharply, entering the cul-de-sac. Finally, the lateral rectal attachments between the distal rectum and distal pelvic side wall, including the levator ani muscle, were taken down with cautery until reaching the level of the peritoneal reflection. The dissection then extended above the reflection as much as possible using LigaSure (Medtronic, Minneapolis, MN, USA) (Figure 2).

#### 2.1.2. Abdominal Phase

We placed a single port for robotic instruments (Da Vinci Single-Port System, Intuitive Surgical, Sunnyvale, CA, USA) at the lower right quadrant site, which was pre-planned for defunctioning the stoma. This 2.5 cm multi-channel port comprises a camera and three interchangeable arms (e.g., scissors, clippers, graspers, etc.).

The stoma aperture was created in the previously marked site, usually in the lower right quadrant: an assistant AirSeal port, 8 mm, was placed below the stoma aperture. The robotic SP (Da Vinci Single-Port System, Intuitive Surgical, Sunnyvale, CA, USA) wound protector was applied, where the outer ring was attached to the sealed tube/bag to contain the SP cannula. Subsequently, the robot was docked, and the instruments were assembled, with scissors in arm 1 and a fenestrated bipolar in arms 2 and 3. The camera was assembled in its channel of a single port. The port was introduced into the abdominal cavity through the stoma aperture, directed toward the left lower quadrant, after the abdomen was insufflated. Once the SP port was in the abdominal cavity, the instruments were extended through the channels.

**Figure 3:**
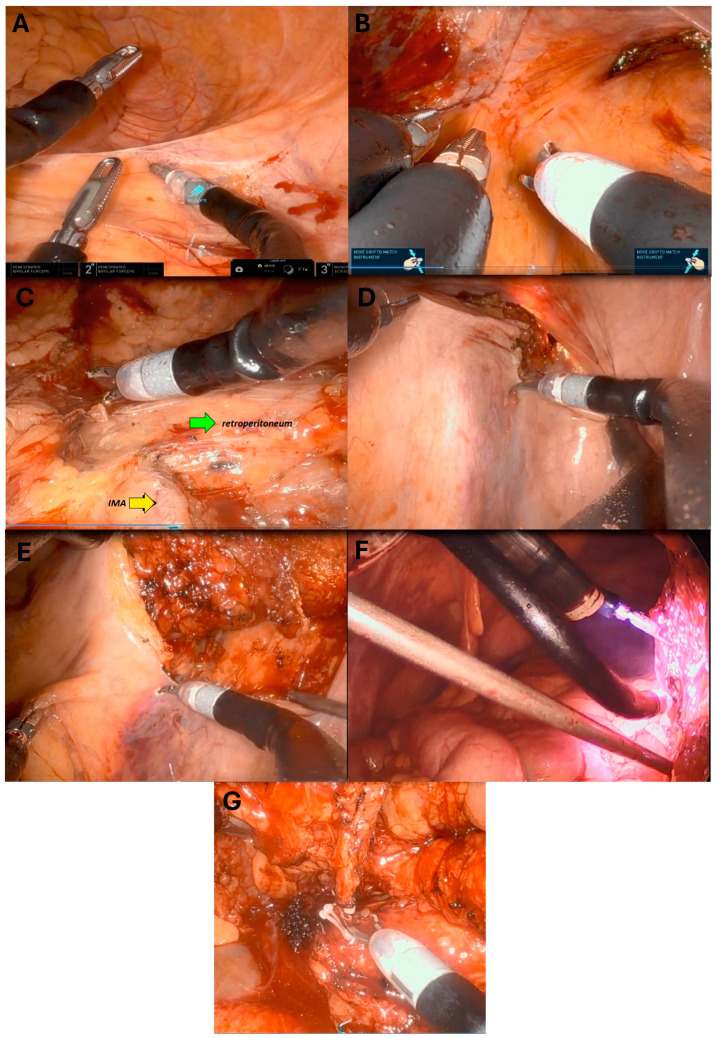
(**A**) Mobilization of the left colon from lateral to medial. (**B**) Dissection of the rectosigmoid junction. Note the upper left incision depicting the upper extent of the trans-anal dissection. (**C**) Full medialization of the left colon showing the retroperitoneum (Green arrow) and the IMA (Yellow arrow) from the lateral aspect. (**D**) Commencing the medial to lateral approach to connect with the lateral dissection. Again, note the extension of the trans-anal dissection till the pararectal gutter. (**E**) Dissection from medial to lateral continuing till the IMA. (**F**) Shows the SP robotic arms while in action dissecting the IMA. (**G**) the IMA is divided using wick clips.

The sigmoid and descending colon were typically mobilized from lateral to medial, up to and around the splenic flexure. Alternatively, a sub-inferior mesenteric vein approach was implemented to mobilize the splenic flexure. Once full medialization was accomplished, identified by reaching the midline, a medial-to-lateral approach was followed at the level of the rectosigmoid junction. This connected with the TaTME dissection plane in the upper pelvis and the lateral dissection plane. The superior hemorrhoidal vessels were identified and followed cephalad until the inferior mesenteric artery was identified. The artery was then skeletonized and secured with clips or an energy device introduced through the assistant port since there was no vessel sealer available for the SP robotic platform at the time. The inferior mesenteric vein was then divided using an energy device, followed by division of the left colic vessel. Then, the mesentery was dissected toward the future transection points in the colon. This was divided similarly until reaching the colonic wall. If the transanal approach failed to reach the upper sacrum, the superior hemorrhoidal vessels were again followed, but this time caudally, entering the TME plane. The final few centimeters of attachments between the upper rectum and the perirectal gutter were then dissected. Hemostasis was also performed once the dissection was deemed complete and tension-free reach was ensured. The robotic instruments were removed, and the robot was undocked (Figure 3).

#### 2.1.3. Transanal Extraction and Anastomosis

In eight cases, the specimen was retrieved transanally without disruption, with a wound protector placed through the anal canal and residual rectum. The colon was transected, and the anvil was secured in the usual fashion. A silk suture (Ethicon Inc., Somerville, NJ, USA) was tied around the anvil to be able to pull on it while creating the end-to-end anastomosis. Then, the anvil with the colonic conduit was placed just beyond the rectal cuff, which was dissected from the surrounding structure, creating a lip in preparation for the second purse-string. Using 0 Prolene (Ethicon Inc., Somerville, NJ, USA), the second purse-string was placed. The anvil was then pulled through the rectum using the silk suture on the anvil with the guidance of a pick-up. The rectal purse-string was then tied tightly around the anvil while pulling on the silk suture to ensure appropriate opposition between the anvil inside the colon and the rectal cuff. The access channel was then removed, and the EEA spike was engaged with the anvil. The surgeon gloved index finger swept around the stapler to make sure no other structure was involved with the stapler interface. The stapler was then fired, and moved, and the donuts were checked for completeness.

**Figure 4:**
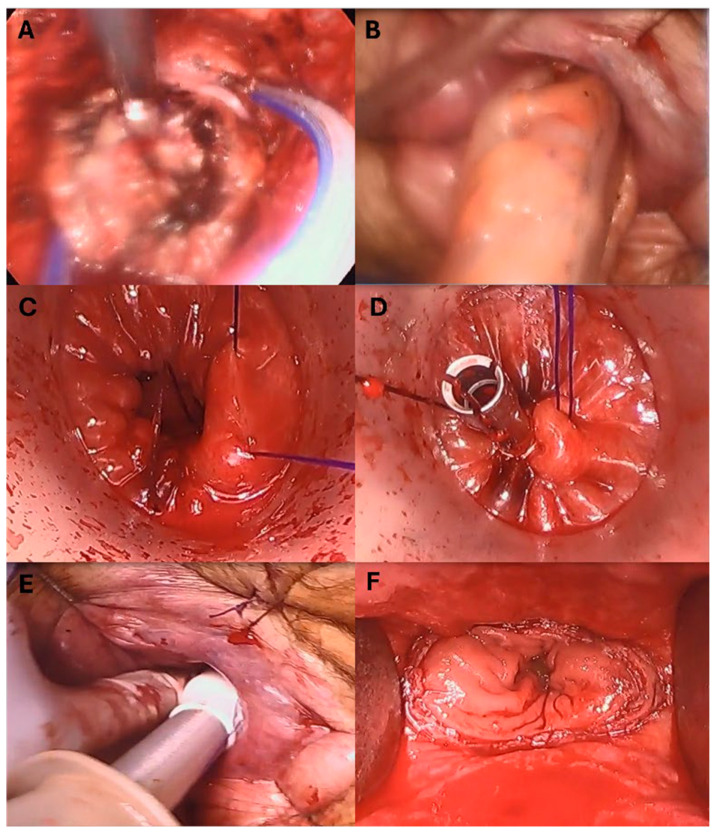
Extraction and anastomosis. (**A**) The drain is placed throughout the abdominal assistant 8 mm port and placed in the pelvis. The specimen is grasped by a Maryland and pulled towards the anus to be retrieved externally. (**B**) The colon is retrieved through the anus. (**C**) Second pursestring is placed using 0 Prolene suture on SH needle (Ethicon Inc., Somerville, NJ, USA). (**D**) The pursestring is tied around the anvil which is pulled through the rectum using the silk suture. (**E**) The EEA spike is engaged with the anvil and surgeon ensuring safe opposition without involving any surrounding structures. (**F**) Anastomosis completed.

In the two cases where the specimens were exteriorized through the stoma site, the colonic conduit was directed to the pelvis after insufflating the abdominal cavity. It was then retrieved transanally using a grasper through the anus after placing the second purse-string suture. The rest of the anastomosis was performed in a similar fashion (Figure 4).

#### 2.1.4. Stoma Creation

A loop of the bowel was chosen to create the diverting loop ileostomy. A 19-French channel Blake drain (Ethicon Inc., Somerville, NJ, USA) was placed through the assistant port into the pelvis. The abdomen was then deflated while the loop of the bowel was held in an oriented fashion and brought through the stoma aperture. The wound protector was removed, and the stoma aperture was reduced in size to accommodate the loop of the bowel, accordingly, using 0 Vicryl (Ethicon Inc., Somerville, NJ, USA) on a UR6 needle. The ileostomy was then sutured to the skin in the usual fashion using 3–0 Vicryl (Ethicon Inc., Somerville, NJ, USA) sutures.

In situations where the specimen could not be accommodated through the anal canal, the specimen was exteriorized through the stoma aperture if deemed appropriate. In our cases, only two specimens, both from en-bloc TAH, were exteriorized through the stoma. After transection and securing the anvil in the colonic conduit, the colon was placed in the abdominal cavity. Insufflation was established, and the conduit was delivered to the distal pelvis in an oriented manner, while the transanal team retrieved it under vision. The anastomosis was then performed as described above.

Postoperative care was provided as per institutional protocols, according to individual needs.

## 3. Results

Ten consecutive patients who had rSPa TaTME dissection for rectal cancer resection were included in the analysis (six females and four males). All ten surgeries were performed consecutively, with eight conducted by one senior surgeon and the remaining two by the same senior surgeon alongside another senior surgeon. The median age at the time of surgery was 53 (range: 37–85), with a mean body mass index (BMI) of 26 kg/m^2^ (Table 1).

One patient (P1) was diagnosed with clinical stage I and went directly to surgery, followed by adjuvant chemotherapy. Of the three patients with clinical stage II, two (P3 and P10) were treated with neoadjuvant short-course radiation, followed by surgery, while the third patient (P5) went straight to surgery. The six patients with clinical stage III underwent total neoadjuvant therapy. Based on pre-operative MRI, the mean distance of the distal tumor border from the anorectal junction was 3.2 cm (range: 2–5.3 cm). All operations were completed as rSPa TaTME without any conversions to a different approach. The average operative time was 351 min. All patients had negative distal and circumferential margins. There were eight complete TME specimens, one near-complete specimen, and one incomplete specimen. The mean number of lymph nodes harvested was 24, and the lowest number of harvested lymph nodes was 13 (Table 2 and Table 3). Nine patients underwent ileostomy reversal. One patient did not undergo ostomy reversal due to functional decline after a stroke. The mean follow-up period was 20 months (4 to 30 months). Six patients experienced complications within 30 days post-surgery, including ileus, small bowel obstruction, acute kidney injury (AKI), deep vein thrombosis, anemia requiring transfusion, and stroke (Table 3). Five of our patients were classified as Clavien–Dindo grade I-II, one patient (P6) as Clavien–Dindo grade IIIA, and the remaining patients as Clavien–Dindo grade 0. Notably, none of our patients experienced symptoms of LARS or a significant increase in stool frequency after stomal reversal.

## 4. Discussion

Rectal cancer resection has witnessed advancement in surgical approaches and minimally invasive modalities, particularly with the emergence of robotic surgery and TaTME, both of which have facilitated surgical techniques for mid- and low-rectal cancers [8,27,28]. TaTME offers a “bottom-up” approach that provides surgeons with improved visualization and access to the distal rectum, addressing some of the limitations inherent in traditional methods, particularly in narrow pelvises. This technique has been successful in oncological outcomes, with studies showing at least equivalent resection margins and reduced local recurrence rates compared to conventional methods [29,30,31,32]. It is essential to remember that TaTME requires specialized training/expertise and is not universally available [33,34]. Single-port approaches to abdominal surgery have benefited patients in terms of omitting multiple incisions and reducing pain. In this series, we have combined these two innovative approaches to rectal cancer resection with rSPa TaTME.

In our initial experience, ten patients successfully underwent the rSPa TaTME approach, with no conversions to a different approach. Postoperative complications were consistent with those typically encountered for proctectomy, including venous thromboembolic events treated with anticoagulation, an abscess requiring antibiotics, a seroma that was drained, ileus or obstruction in three patients managed conservatively, a UTI requiring antibiotics, AKI managed with hydration, and anemia requiring transfusion. One patient did experience a stroke and did not have their defunctioning ileostomy reversed, while the remaining nine patients had their gastrointestinal continuity restored. The oncologic outcomes were reassuring, with eight complete TME specimens, one nearly complete, and one incomplete. All had adequate lymph node retrieval, with the lowest number being 13. With a mean follow-up of 20 months, no patient experienced cancer recurrence. At centers with robotic surgery and TaTME expertise, rSPa TaTME offers an innovative approach to distal rectal cancers. It is imperative to mention that the mean operative time was more than 5 h since most of these cases were performed consecutively, in addition to getting used to the SP ergonomics. These ergonomics are entirely different than the straight sticks and XI multi-port robotic platform. Unlike XI instruments, where 3-D movement is available at the used tip (scissors, fenestrated bipolar, etc.), the SP arms have an elbow that bends acutely, affecting the trajectory of instrument movement.

Furthermore, handling the camera with and without the cobra position is not initially easy. The cobra position is obtained by bending the camera shaft at its elbow, thus providing a semi-overview of the dissection. Finally, moving inside the abdominal cavity requires full pivot-like movement. This requires retracting the instruments in their respective channels, moving the port toward the desired location, and then reintroducing them accordingly. Collectively, this resulted in longer operative durations, in addition to the usual factors, such as visceral obesity and adhesions.

### Limitations

The retrospective nature of our small cohort of patients presents limitations in the level of evidence. The case series nature of our study limits its comparability to other techniques. More extensive studies are required.

## 5. Conclusions

Combining the abdominal single-port robotic approach with transanal total mesorectal excision (rSpa TaTME) for the resection of distal rectal cancer showed acceptable surgical and oncologic outcomes in this initial series. However, this technically demanding procedure is still being explored and should be performed in high-volume centers, with appropriate experience and careful review.

## Figures and Tables

**Table 1:** Patient demographics.

	Patient Number (*n* = 10)
Biological Sex	
Male	4
Female	6
Race	
White (non-Hispanic)	8
Asian (Filipino)	1
Unknown	1
Age at surgery, mean (SD)	53 (±13)
BMI (kg/m²), mean (SD)	26 (±5)
ASA, (%)	
I	1
II	8
III	1

SD: standard deviation; BMI: body mass index (kg/m²); ASA: American Society of Anesthesiologists.

**Table 2:** Tumor characteristics and treatment.

Case	cT *	cN	Clinical Stage	High-Risk Features	Tumor Size (cm)	Distance from ARJ (cm)	Neoadjuvant Therapy	Adjuvant Therapy
P1	2	0	I	PNI +, LVI +	3.3	2.0	-	Chemotherapy
P2	4	2	IIIC	MRF +	4.8	3.0	TNT	-
P3	3	0	IIA	-	2.8	3.9	SCRT	-
P4	3	1	IIIB	EMVI +	2.5	2.0	TNT	-
P5	3	0	IIA	-	2.5	6.5	-	-
P6	3	1	IIIB	EMVI +	3.6	3.2	TNT	-
P7	3	1	IIIB	-	9.5	7.0	TNT	-
P8	3	1	IIIB	-	5.4	4.0	TNT	-
P9	2	1	IIIA	-	1.9	5.0	TNT	-
P10	3	0	IIA	EMVI +, PNI +, LVI +	3.0	1.1	SCRT	-

cT: clinical tumor; cN: clinical node; P: patient; MRF: mesorectal fascia; EMVI: extramural venous invasion; LVI: lymphovascular invasion; PNI: perineural invasion; ARJ: anorectal junction; TNT: total neoadjuvant therapy; SCRT: short-course radiotherapy. * All patients were M0.

**Table 3:** Operative and postoperative outcomes (tumor).

Case	Operative Time (min)	EBL	Concurrent Procedures	LOS (Days)	Margins (CRM, DM)	TME Grade	LN Harvest	Postoperative Complication Within 60 Days	Stoma Reversed	Follow-up (Months)
P1	252	300		3	Negative	Complete	51	DVT	Yes	30
P2	426	500	TAH BSO	7	Negative	Complete	18	Anemia requiring transfusion, UTI, stroke	No	30
P3	295	10	-	3	Negative	Near complete	32	-	Yes	27
P4	204	100	-	2	Negative	Complete	20	-	Yes	28
P5	371	100	-	3	Negative	Complete	28	Abscess (antibiotics), SBO	Yes	15
P6	332	350	-	2	Negative	Complete	13	Pelvic seroma (drain), and IMV thrombus	Yes	21
P7	215	25	-	3	Negative	Complete	15	-	Yes	19
P8	406	200	TAH BSO	2	Negative	Complete	17	SBO	Yes	15
P9	372	100	-	7	Negative	Incomplete	23	-	Yes	4
P10	465	31	-	11	Negative	Complete	25	Ileus, AKI	Yes	9

P: patient; EBL: estimated blood loss; LOS: length of stay; CRM: circumferential margin; DM: distal margin; TME: total mesorectal excision; LN: lymph node; DVT: deep vein thrombosis; TAH BSO: total abdominal hysterectomy with bilateral salpingo-oophorectomy; UTI: urinary tract infection; SBO: small bowel obstruction; IMV: inferior mesenteric vein; AKI: acute kidney injury.

## Data Availability

The data supporting the findings of this study are not publicly available but are available upon reasonable request from the corresponding author with appropriate justification.

## Abbreviations

TaTME	transanal total mesorectal excision
rSPa	single-port robotic abdominal
SP	single port
TME	total mesorectal excision
SD	standard deviation
BMI	body mass index
P	patient
cT	clinical tumor
N	node
MRI	magnetic resonance imaging
AKI	acute kidney injury
ASA	American Society of Anesthesiologists
cN	clinical node
MRF	mesorectal fascia
EMVI	extramural venous invasion
LVI	lymphovascular invasion
PNI	perineural invasion
ARJ	anorectal junction
TNT	total neoadjuvant therapy
SCRT	short-course radiotherapy
EBL	estimated blood loss
LOS	length of stay
CRM	circumferential margin
DM	distal margin
LN	lymph node
CVT	deep vein thrombosis
TAH BSO	total abdominal hysterectomy with bilateral salpingo-oophorectomy
UTI	urinary tract infection
SBO	small bowel obstruction
IMV	inferior mesenteric vein
